# Hypoxia blocks ferroptosis of hepatocellular carcinoma via suppression of METTL14 triggered YTHDF2‐dependent silencing of SLC7A11

**DOI:** 10.1111/jcmm.16957

**Published:** 2021-10-05

**Authors:** Zhuoyang Fan, Guowei Yang, Wei Zhang, Qian Liu, Guangqin Liu, Pingping Liu, Ligang Xu, Jianhua Wang, Zhiping Yan, Hong Han, Rong Liu, Minfeng Shu

**Affiliations:** ^1^ Department of Interventional Radiology Zhongshan Hospital Fudan University Shanghai China; ^2^ Department of Pharmacology School of Basic Medical Sciences Shanghai Medical College Fudan University Shanghai China; ^3^ Department of Ultrasound Zhongshan Hospital of Fudan University Shanghai China; ^4^ Department of Interventional Radiology Zhongshan Hospital Fudan University Shanghai China; ^5^ Department of Interventional Radiology Xiamen Branch Zhongshan Hospital Fudan University Shanghai China

**Keywords:** ferroptosis, hepatocellular carcinoma, hypoxia, METTL14, SLC7A11

## Abstract

Residue hepatocellular carcinoma (HCC) cells enduring hypoxic environment triggered by interventional embolization obtain more malignant potential with little clarified mechanism. The N^6^‐methyladenosine (m^6^A) biological activity plays essential roles in diverse physiological processes. However, its role under hypoxic condition remains largely unexplored. RT‐qPCR and Western blot were used to evaluate METTL14 expression in hypoxic HCC cells. MDA assay and electronic microscopy photography were used to evaluate ferroptosis. The correlation between SLC7A11 and METTL14 was conducted by bioinformatical analysis. Flow cytometry was used to verify the effect of SLC7A11 on ROS production. Cell counting kit‐8 assay was performed to detect cells proliferation ability. Hypoxia triggered suppression of METTL14 in a HIF‐1α–dependent manner potently abrogated ferroptosis of HCC cells. Mechanistic investigation identified SLC7A11 was a direct target of METTL14. Both in vitro and in vivo assay demonstrated that METTL14 induced m^6^A modification at 5’UTR of SLC7A11 mRNA, which in turn underwent degradation relied on the YTHDF2‐dependent pathway. Importantly, ectopic expression of SLC7A11 strongly blocked METTL14‐induced tumour‐suppressive effect in hypoxic HCC. Our investigations lay the emphasis on the hypoxia‐regulated ferroptosis in HCC cells and identify the HIF‐1α /METTL14/YTHDF2/SLC7A11 axis as a potential therapeutic target for the HCC interventional embolization treatment.

## 1 INTRODUCTION

Liver cancer is one of the most common and aggressive tumours that causes 841,000 new cases and 782,000 deaths each year in the world.^1^ Hepatocellular carcinoma (HCC) is the most common primary liver cancer characterized by rapid proliferation and metastasis. Both the Barcelona Clinic Liver Cancer (BCLC) criteria and Guidelines (2017 Edition) for Diagnosis and Treatment of Primary Liver Cancer in China have recommended proper means to treat HCC at different stages.^2^, ^3^ Multiple approaches of treatment are still needed in clinic since the disease is generally diagnosed at the advanced stage^4^ and the 5 years survival rate of HCC patients remains unsatisfactory.^5^

Interventional therapy has been widely applied to patients with unresectable HCC, whereas the hypoxic condition caused by interventional embolization could benefit HCC cells a lot for proliferation and metastasis,^6^, ^7^, ^8^ and the underlying mechanism remains unclear. Therefore, exploring the molecular mechanisms by which hypoxic HCC develops is critical to advance future therapeutic strategies. Strikingly, it has been demonstrated that the aberrant epigenetic changes can result in profound disruption of gene expression to facilitate HCC initiation and progression.^9^

N^6^‐Methyladenosine (m^6^A) RNA modification has become a new dimension of epigenetic regulatory mechanism that controls mRNA expression before translation. m^6^A was found in at least one‐third of the mammalian mRNAs. It is estimated that there is an average of 3 to 5 m^6^A modifications in one mRNA. Notably, many m^6^A sites are evolutionally conserved between mice and human beings. A multi‐component m^6^A methyl transferase complex (MTC) executes the deposition of m^6^A in mRNAs modifications, which consists of a heterodimer, the methyltransferase‐like 3 (METTL3)/methyltransferase‐like 14 (METTL14) complex, the major enzymatic complex and other participating factors including KIAA1429, WTAP and RBM15.^10^, ^11^, ^12^, ^13^, ^14^ In terms of this complex, METTL3 is the catalytic subunit that binds to the methyl donor S‐adenosyl methionine (SAM) and catalyses methyl group transfer, whereas METTL14 is responsible for m^6^A deposition by stabilizing METTL3 conformation and recognizing substrate RNAs.^15^, ^16^ The m^6^A modification sites, which are frequently enriched in the 3’ untranslated region (3’ UTR) and coding sequence (CDS), with a particularly high enrichment around the stop codon area contain a classical consensus sequence DRACH (D = G, A, or U; R = G or A; H = A, C, or U).^17^, ^18^ This reversible catalytic process is carried on by m^6^A eraser fat mass and obesity‐associated protein (FTO) and alkB homologue 5 (ALKBH5).^19^, ^20^ m^6^A‐modified RNA can be identified by m^6^A reader proteins, including YT521‐B homology (YTH) domain family proteins (YTHDF1–YTHDF3, YTHDC1 and YTHDC2).^17^, ^21^, ^22^ Among them, YTHDF2 is the first identified and well‐studied m^6^A reader protein which targets m^6^A via its C‐terminal YTD domain and the N terminal domain is responsible for pushing the mRNA to the processing bodies for further degradation.^22^

Recent studies suggest that m^6^A is involved in diverse physiological processes.^23^, ^24^, ^25^ As another indispensable component of the MTC, METTL14 has been shown to be upregulated in various types of cancer. Weng et al.^26^ reported knockdown of METTL14 could significantly inhibit self‐renewal of leukaemia stem/initiating cells (LSCs/LICs). Further mechanistic studies revealed that the decrease of m^6^A abundance on its target mRNAs MYB and MYC led to reduction of the mRNA stability and translation. METTL14 was also reported to drive EBV‐mediated tumorigenesis.^27^ However, Chen et al. found that METTL14 suppressed colorectal cancer (CRC) progression via targeting miR‐375^28^;Ma et al. held the opinion that METTL14 inhibited HCC metastasis via targeting pri‐miR‐126.^29^ Therefore, the concrete function of METTL14 in tumorgenicity remains elusive.

Ferroptosis which is mainly characterized by the iron‐dependent induction of lipid peroxidation products and/or lethal reactive oxygen species (ROS) is a recent identified non‐apoptotic cell death, showing a tremendous promising in cancer therapy.^30^ The function of system x_c_^−^ which is made of SLC7A11 and SLC3A2 is to import the extracellular oxidized form of cysteine, cystine and export intracellular glutamate out to keep the balance of Redox.^31^, ^32^ Higher level of SLC7A11 has been detected in several types of human cancer, and inhibition of SLC7A11 would render tumour cells susceptible to ferroptosis.^33^ Ungard et al. found silence of SLC7A11 in breast cancer cells delayed onset of cancer‐induced bone pain^34^; Lee et al. gave a prediction that overexpression of SLC7A11 had a positive correlation of recurrence in patients with oral cavity squamous cell carcinoma.^35^ All these data suggest that system x_c_^−^ inhibitors may work as potential promising anti‐cancer agents. However, our knowledge about the exact mechanisms by which regulation of ferroptosis in HCC, especially under hypoxia condition remains unclear. Whether disruption of the m^6^A machinery could result in ferroptotic cell death and contribute to HCC pathogenesis still needs to be explored.

In this study, by utterly investigating the alteration of METTL14 under hypoxia and the function of upregulated METTL14 in HCC pathogenesis, we found that METTL14 controlled ferroptosis by inducing ROS release and negatively regulating SLC7A11 expression via triggering m^6^A methylation at 5‘UTR of SLC7A11 mRNA. Meanwhile, overexpression of SLC7A11 could significantly rescue METTL14‐induced tumour‐suppressive effect under hypoxia in HCC. Our study indicates that targeting ferroptosis via METTL14/YTHDF2/SLC7A11 axis might be a potential therapeutic approach for advance the HCC interventional embolization treatment.

## 2 METHODS

### 2.1 Cell culture and treatment

As the previous study, we performed cell culture and treatment.^36^ Briefly, seven human hepatic malignant cell lines (Huh7, HepG2, 7721, HCCLM3, MHCC97H, PLC/PRF/5 and Bel‐7402) and one normal human liver cell line L02 were obtained from Dr. Shizhe Zhang and Dr. Wei Gan, Zhongshan Hospital, Fudan University. All cell lines were cultured in Dulbecco's modified Eagle's medium (DMEM, Gibco, Grand Island). The extra mixture of DMEM is 10% foetal bovine serum (FBS), antibiotics (penicillin (100 U/ml)/streptomycin (0.1 mg/ml)). A humidified environment with 5% CO_2_ and 37℃ is needed to cultivate the cells. The medium was refreshed twice a week. N‐acetyl‐L‐cysteine (NAC) was purchased from Apexbio. We set 1% oxygen as the condition of hypoxia if the experiments were needed.

### 2.2 Transfection and stable cell lines

We manipulated transfection and stable cell lines referring to the previous study.^37^ Lipofectamine 2000 Reagent (Life Technology, Thermo Fisher Scientific) was used to perform the transient transfection. pcDNA3.1‐METTL14, pcDNA3.1‐YTHDF2, pLKO.1‐SLC7A11 and pcDNA3.1‐SLC7A11‐His(+) were obtained from Genechem. An empty vector was used as negative control. The transfection assays using Lipofectamine 2000 Reagent (Invitrogen) were performed according to the manufacturer's instructions. A total of 5 × 10^5^ cells were used for this experiment. After 48 h of transfection, transfection efficiency was validated by RT‐qPCR analysis or Western blot. The scrambled non‐target shRNA (NC): TTCTCCGAACGTGTCACGTTTC; shSLC7A11: GCAGCTACTGCTGTGATATCC.

### 2.3 Quantitative real‐time PCR

The quantitative real‐time PCR is carried out as described before.^36^ According to the manufacturer's instructions, total RNA was extracted by RNA Purification Kit (EZbioscience). Afterwards, 4× Reverse Transcription Master Mix (EZbioscience) was used for RT‐qPCR with DNase‐free and RNase‐free tips (YueYiBioTech). A SYBR Green PCR kit (Yeasen) was used for qPCR. The expression level of each gene was normalized to that of GAPDH, which served as an internal control according to the 2^−ΔΔCt^ method. Primers (Sunya, China) for METTL14, YTHDF2, SLC7A11, E‐cadherin, N‐cadherin, Vimentin and GAPDH were as follows: METTL14 (F: 5'‐ CATCAGGCTAAAGGATGAGTT‐3'; R: 5'‐ CTAACTTCATAATATCATCC‐3'), YTHDF2(F:5'‐AGCCCCACTTCCTACCAGATG‐3'; R: 5'‐ TGAGAACTGTTATTTCCCCATGC‐3'),SLC7A11 (F: 5'‐GTCTGGAGAAACAGCCAAGG‐3'; R: 5'‐CGGAGTTCCTCGAATAGCTG‐3'), E‐cadherin (F: 5'‐ CGAGAGCTACACGTTCACGG‐3'; R: 5'‐ GGGTGTCGAGGGAAAAATA GG‐3'), N‐cadherin (F: 5'‐ CTGACAATGACCCCACAGC‐3'; R: 5'‐TCCTGCTCACCACCACT ACTT‐3'), Vimentin (F: 5'‐ TCTACGAGGAGGAGATGCGG‐3'; R: 5'‐GGTCAAGACGTGCCAG AGAC‐3'),and GAPDH (F: 5'‐GCACCGTCAAGGCTGAGAAC −3'; R: 5'‐TGGTGAAGAC GCCAGTGGA‐3').

### 2.4 Cell proliferation assay

Cell proliferation was detected by an indirect method, Cell Counting Kit‐8 assay (Yeason) according to the manufacturer's protocol. In each well, 10,000 cells were planted for 24 h or 12 h according to the manufacturer's protocol. At the end of the checking point, 10 μl of CCK8 solution was used to detect the OD value of each well. The solution was measured spectrophotometrically at 450 nm.

### 2.5 Western blot

The Western blot is performed as previous work.^38^ In details, cells were harvested by RIPA, and 20 µg of total protein of each group was used for electrophoresis. The membrane was incubated with 5% nonfat milk at 22°C for 2 h and then incubated with primary antibodies for overnight at 4°C. The major antibodies and their dilutions are as follows:

METTL14 (D8K8W, CST): Dilution‐1: 1000.

HIF‐1α (D2U3T, CST): Dilution‐1: 500.

Actin (13E5, CST): Dilution‐1: 1000.

SLC7A11 (ab175186, abcam): Dilution‐1: 1000.

COX2 (D5H5, CST): Dilution‐1: 1000.

YTHDF2 (ab220163, abcam): Dilution‐1: 1000.

E‐cadherin (24E10, CST): Dilution‐1: 1000.

N‐cadherin (D4R1H, CST): Dilution‐1: 1000.

Vimentin (D21H3, CST): Dilution‐1: 1000.

GAPDH (14C10, CST): Dilution‐1: 1000.

Anti‐rabbit IgG, HRP‐linked Antibody (#7074, CST): 1: 3000.

Subsequently, an anti‐rabbit secondary antibody was used for 2 h at 22°C (about room temperature). The protein bands were visualized using a chemiluminescence ECL kit (Tanon).

### 2.6 Wound healing assay

The wound healing assay is followed with the previous work.^39^ Briefly, in order to evaluate the migration ability of cells, wound healing assays were performed. NAC and/or pcDNA3.1‐SLC7A11‐His(+) (2 µg) were transfected to the wells if needed. The cells were incubated at the condition of 37℃ and 5% CO_2_. A scratch was performed by a 20–200 µl pipet tip, and subsequently, the plate was washed with PBS and replaced with fresh medium. Wound healing images were taken at 0 and 48 h after scratching using a phase‐contrast microscope (Canon).

### 2.7 ROS detection

Our ROS detection was validated by Flow cytometer similar to a previous work.^40^ Briefly, Oxygen Species Assay Kit was purchased from Yeasen. Intracellular ROS levels were measured by flow cytometry using the cell‐based ROS assay kit according to the manufacturer’s instructions. As the protocol, after interfered by NAC (if needed), the cells were digested by trypsin and washed with PBS. 10 μM DCFH‐DA was used for detection in DMEM for 30 min at 37°C. The fluorescence was measured by flow cytometry. The excitation wavelength was 488 nm, and the emission wavelength was 530 nm. For each analysis, 10,000 cells were recorded. Intracellular ROS levels were expressed as the average DCF fluorescence intensity of the cells.

### 2.8 Dual‐Luciferase reporter assay

The dual‐luciferase reporter assay was measured like the previous study.^39^ Plasmids were constructed in advance. Briefly, procedures were followed to the manufacturer's instruction (11402ES60, Yeasen). Huh7 or HCCLM3 cells were plated in 6‐well plates and transfected with the wide‐type SLC7A11‐responsive luciferase reporter construct (SLC7A11‐WT), the mutant SLC7A11‐responsive luciferase reporter construct (SLC7A11‐MUT), the wide‐type METTL14 plasmids or the siYTHDF2, accordingly. At 24 h post‐transfection, cell lysates were incubated with 10 μg/ml of firefly and TK separately for 10 min. The luciferase activities were measured by Dual‐Luciferase Reporter Assay System (Promega, Madison) and a microplate luminometer (Promega). The firefly luciferase activities were corrected by the corresponding renilla luciferase activities. The wide‐type and mutated promoter sequence of SLC7A11 were provided in supplemental materials (S1).

### 2.9 Methylated RNA immunoprecipitation (MeRIP)

MeRIP was followed by the manufacturer's instruction (Pierce™ Magnetic RNA‐Protein Pull‐Down Kit, Cat. No. 20164). Briefly, 200 μg of total RNA was isolated for polyA^+^ RNA (Promega) and quantified. PolyA^+^ RNA was fragmented to approximately 100 nt long fragments. Before proceeding to m^6^A‐IP, RNA fragmentation was ensured by using a bioanalyzer. Afterwards, first‐strand cDNA synthesis was performed first. qPCR was performed as described.

### 2.10 RNA decay assay

In the RNA decay assay, we mainly followed the method used by Qing et. al.^41^ Briefly, Huh7 and HCCLM3 cells upon METTL14 or YTHDF2 were treated with 3 mg/ml actinomycin D (A9415, Sigma‐Aldrich). In order to see the RNA stability of SLC7A11, cells were treated as described in method and harvested at the certain time points (8 h, 4 h, 2 h and 0 h). In addition then, the total RNA was extracted, with the DNase‐I digesting to eliminate genomic DNA. Afterwards, the collected RNA was reversed transcribed into cDNA followed by RT‐qPCR detection. The primer of SLC7A11 used in RT‐qPCR has been described above: SLC7A11 (F: 5'‐GTCTGGAGAAACAGCCAAGG‐3'; R: 5'‐CGGAGTTCCTCGAATAGCTG‐3').

### 2.11 MDA Assay

The MDA assay was gone through in the light of the previous study.^42^ To test the lipid peroxidation, we use Lipid Peroxidation (MDA) Assay Kit (Cat. No. MAK085, Sigma‐Aldrich). The procedure was followed by the manufacturer's instruction.

### 2.12 Electron microscopy photography

The electron microscopy photography was referred to Caroline's study.^43^ To obtain the location of mitochondria in the cells, electron microscopy photography was taken for HCC cells with different intervention. For imaging, the cells were prepared as follows. Samples were fixed by 1% Osmium acid under 4°C for 1 h. Afterwards, ddH_2_O was used to wash the samples. Then, samples were stained by uranium acetate for overnight. Different concentration of alcohol was applied to dehydrate the samples. Samples were embedded by EMBED 812 EMBEDDING KIT and finally were polymerized for imaging.

### 2.13 Animal study

The BALB/C nude mice were obtained from Shanghai SLAC Laboratory Animal Co., Ltd. We performed the nude mice experiment referring to the previous work.^44^ Briefly, for the subcutaneous implantation model, 5 × 10^5^ stable SLC7A11‐knockdown HCCLM3 cells or SLC7A11‐vector cells were injected subcutaneously into BALB/C nude mice. After 5 weeks, mice were killed, and tumour photograph was detected with photography. In the other animal work, 5 × 10^5^ stable METTL14‐vector HCCLM3 cells, SLC7A11‐Overexpression cells or SLC7A11‐R298P cells were injected subcutaneously into BALB/C nude mice. After about 5 weeks, mice were killed, and tumour photograph was detected with photography. The agreement of animal experiments from Animal Center of our unit was provided in the additional files (Additional File 2).

### 2.14 H&E staining and Immunohistochemistry

The H&E staining and Immunohistochemistry was performed as the previous study.^45^ Briefly, xenograft samples were fixed in 4% paraformaldehyde (Sigma‐Aldrich, DK‐2860) overnight and embedded in paraffin before sections of 6 µm were cut. Antigen retrieval was performed at 100°C in a citrate buffer for 25 min. 0.1% Tween20 and 2.5% BSA (Sigma‐Aldrich) was mixed to the samples. Primary antibodies are as follows: METTL14 (ab220030, Abcam), SLC7A11 (ab37185, Abcam) and COX2 (ab179800, Abcam). Proper horseradish‐peroxidase‐conjugated secondary antibodies were applied for development (ab205718, Abcam).

### 2.15 Statistical analysis

In this study, GraphPad Prism 8.00 (GraphPad Software) was performed for analyses. Student's *t* test was used for statistical analysis. A *p* value less than 0.05 was considered statistically significant. Data are presented as the means ± SEM.

## 3 RESULTS

### 3.1 The levels of METTL14, ROS and lipid peroxidation were limited by HIF‐1α in hypoxia condition

**FIGURE 1:**
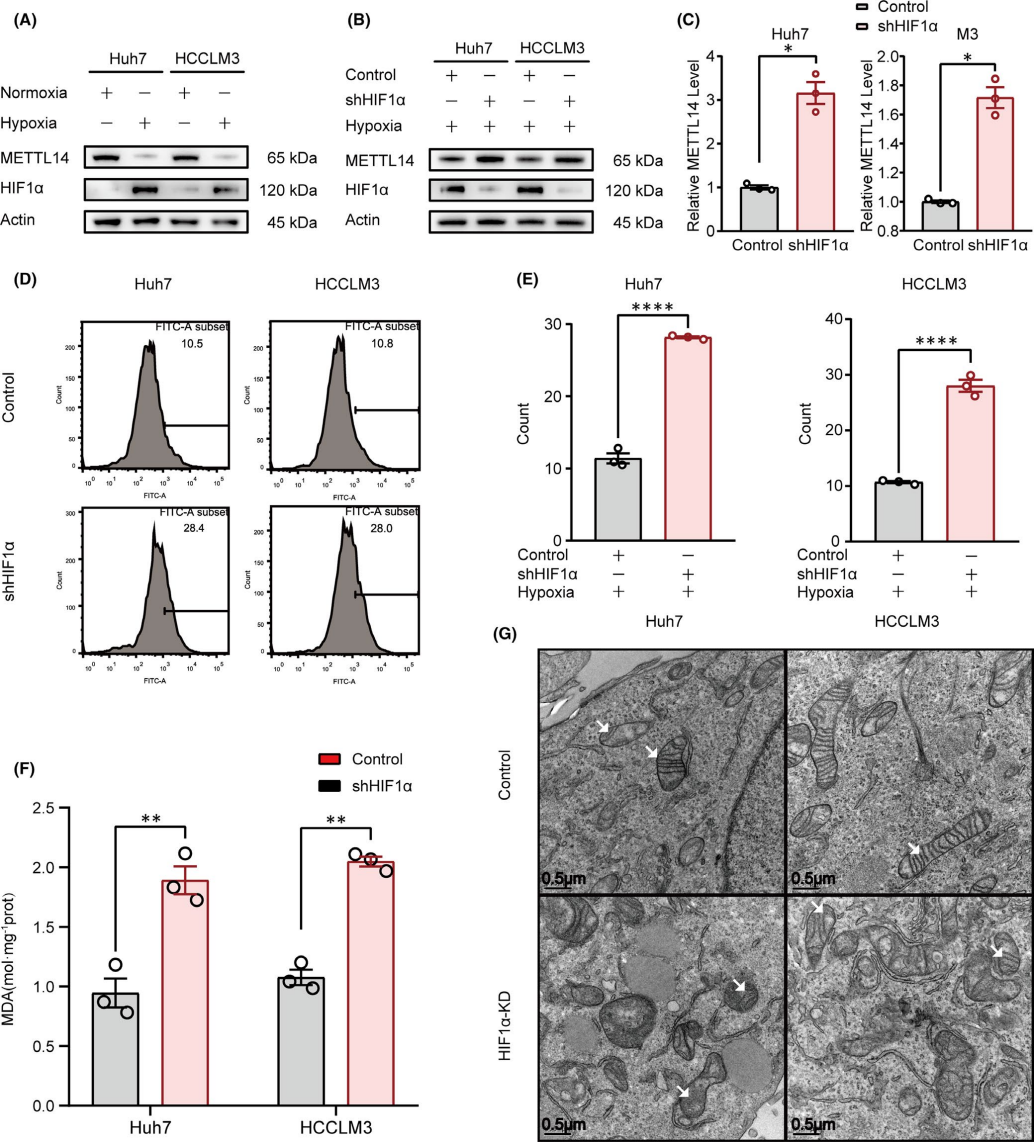
The levels of METTL14, ROS and lipid peroxidation were limited by HIF‐1α in hypoxia condition. (A) Hypoxia downregulated METTL14 in Huh7 and HCCLM3 cells. (B&C). Effect of HIF‐1α knockdown on hypoxia‐induced METTL14 suppression. (D&E) Effect of HIF‐1α knockdown on hypoxia‐induced ROS accumulation detected by flowcytometry (D) and the quantification was also shown (E). F. Effect of HIF‐1α knockdown on hypoxia‐induced MDA accumulation. (G) The morphological change of mitochondria was detected by electronic microscopy. The white arrows referred to typical mitochondria. ‘NS’, not significant, *p < 0.05, **p < 0.01, ***p < 0.001, ****p < 0.0001

To determine the role of METTL14 and RNA m^6^A modification under hypoxia, we first examined the expression of METTL14 in Huh7 and HCCLM3 cell lines with 1% O_2_ (Hypoxic condition). Our data confirmed that compared to control group (Normoxia), hypoxia efficiently decreased the METTL14 and increased the HIF‐1α expression in the two HCC cell lines (Figure 1A). Intriguingly, inhibition of HIF‐1α by shRNA strongly blocked the hypoxia‐induced downregulation of METTL14 (Figure 1B,C), which indicated hypoxia triggered suppression of METTL14 was HIF‐1α dependent. Ferroptosis is a form of regulated cell death and has been demonstrated to play a tumour‐suppressive function that could be harnessed for cancer therapy. ROS and MDA are well‐established indicators of ferroptosis. To explore the effect of hypoxia on ferroptosis, ROS was determined by flow cytometry. Our data showed inhibition of HIF‐1α enhanced accumulation of ROS in both Huh7 and HCCLM3 cell lines (*p* < 0.0001) (Figure 1D,E). What's more, MDA assay revealed that compared with that in control group, MDA contents were higher in HIF‐1α knockdown group (*p* < 0.01) (Figure 1F). Additionally, it is known that ferroptosis could induce typical morphological changes characterized by condensed and disrupted mitochondria.^46^, ^47^ Consistently, we did observe that HIF‐1α knockdown dramatically induced smaller and denser mitochondria with damaged membrane compared to that in control group (Figure 1G). In summary, our data indicated hypoxia downregulated METTL14 in the HIF‐1α–dependent manner and knockdown of HIF‐1α induced ferroptosis in HCC cells.

### 3.2 METTL14 negatively regulates SLC7A11 expression in HCC

**FIGURE 2:**
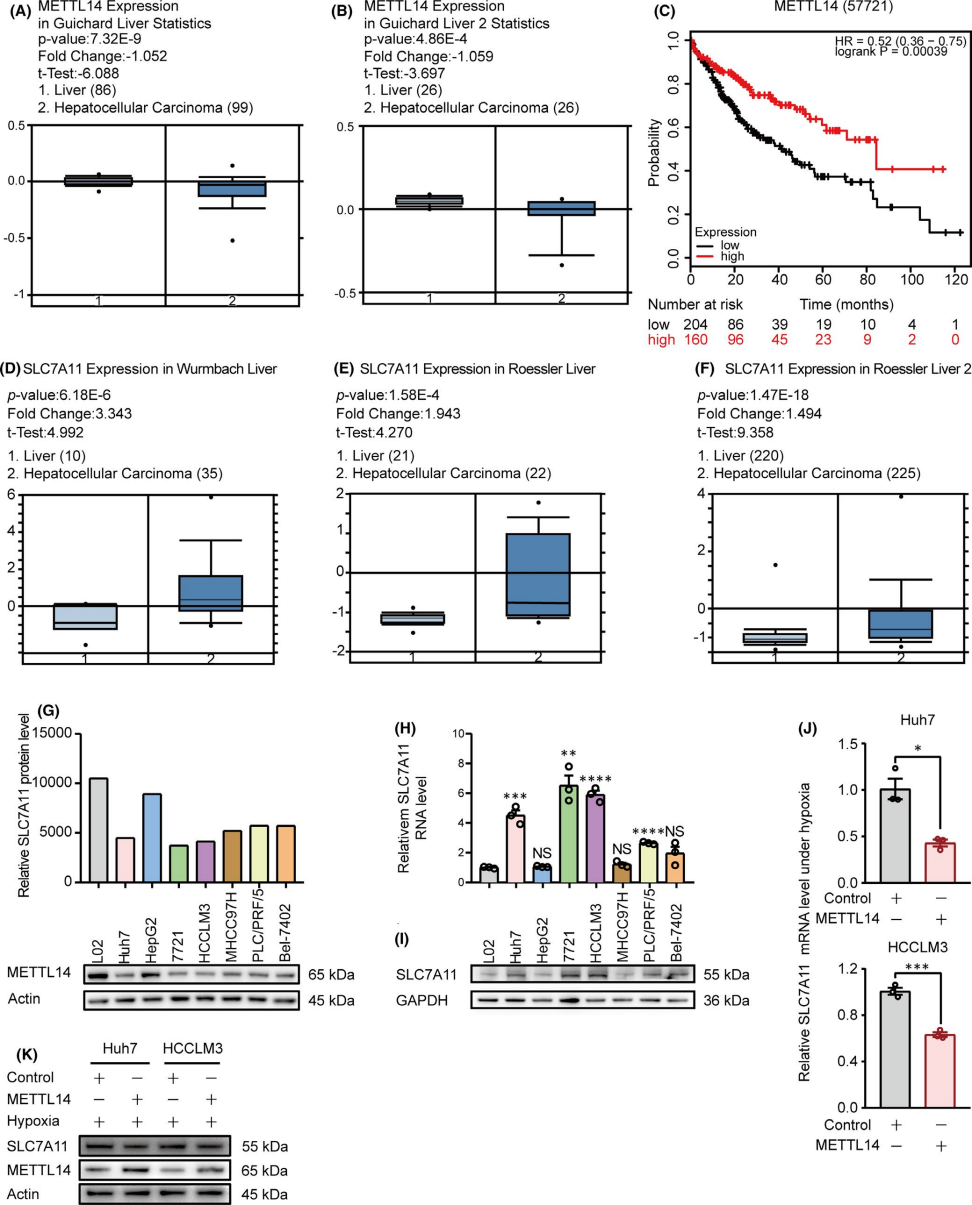
METTL14 negatively regulates SLC7A11 expression in HCC. (A–C) Bioinformatics analysis from ‘Guichard Liver’ and ‘Guichard Liver 2’ database analysed by Oncomine (https://www.oncomine.org/resource/main.html) and TCGA database analysed by OncoLnc (http://www.oncolnc.org/) with the keywords ‘METTL14’, ‘Hepatocellular Carcinoma vs. Normal Analysis’. (D–F) Bioinformatics analysis from ‘Wurmbach Liver’, ‘Roessler Liver’ and ‘Roessler Liver 2’ database analysed by Oncomine (https://www.oncomine.org/resource/main.html) with the keywords ‘SLC7A11’, ‘Hepatocellular Carcinoma vs. Normal Analysis’. (G) The protein expression pattern of METTL14 among seven HCC cell lines (Huh7, HepG2, 7721, HCCLM3, MHCC97H, PLC/PRF/5 and Bel‐7402), comparing to normal liver cell line L02 detected by Western blot (low panel). The relative METTL14 protein level was quantified as showed in up panel. (H&I) The mRNA and protein levels of SLC7A11 among seven HCC cell lines (Huh7, HepG2, SMMC‐7721, HCCLM3, MHCC97H, PLC/PRF/5 and Bel‐7402), comparing to normal liver cell line L02 detected by qPCR and Western blot, separately. (J&K) The effect of METTL14 on SLC7A11 expression in Huh7 and HCCLM3 cells under hypoxia. The SLC7A11 mRNA and protein levels were detected by RT‐qPCR and Western blot, respectively. ‘NS’, not significant, *p < 0.05, **p < 0.01, ***p < 0.001, ****p < 0.0001

To explore the potential mechanism by which METTL14 regulated ferroptosis, the expression pattern of METTL14 and SLC7A11 which is a core member of system x_c_^−^ that mediates ferroptosis was analysed with multiple databases. Firstly, the expression pattern of METTL14 in HCC patients vs the healthy counterpart was analysed in The Cancer Genome Atlas (TCGA) and Oncomine databases. Compared with normal liver, the expression level of METTL14 was up to −1.052‐fold (*p* = 7.32×10^−9^) (Figure 2A) and −1.059‐fold (*p* = 4.86×10^−4^) (Figure 2B) in HCC patients, respectively. On the contrary, the overall survival of METTL14‐lower expressed HCC patients was shorter than that of SLC7A11‐higher expressed counterpart (Figure 2C). Moreover, the expression of SLC7A11 in HCC patients and healthy candidates was further analysed in Oncomine database ‘Wurmbach Liver’, ‘Roessler Liver’ and ‘Roessler Liver 2’. Conversely, compared with normal liver, the expression of SLC7A11 was upregulated to 3.343‐fold (*p* = 6.18 × 10^−6^) (Figure 2D), 1.943‐fold (*p* = 1.58 × 10^−4^) (Figure 2E) and 1.494‐fold (*p* = 1.47 × 10^−18^) (Figure 2F) in HCC patients, respectively.

The METTL14 expression was further analysed in several HCC cell lines. Compared to normal liver cell line L02, the METTL14 protein was decreased in Huh7, 7721, HCCLM3, MHCC97H, PLC/PRF/5 and Bel‐7402 cell lines, except for HepG2 (Figure 2G).

Interestingly, both mRNA and protein level of SLC7A11 was markedly upregulated in most of the HCC cell lines detected, except for HepG2 and MHCC97H (Figure 2H,I), suggesting that METTL4 may reversely correlated with SLC7A11 expression. Importantly, ectopic expression of METTL14 under hypoxia could significantly downregulate SLC7A11 expression at both mRNA and protein level (Figure 2J,K). Taken together, these data indicate that METTL14 negatively regulates SLC7A11 expression.

### 3.3 METTL14 triggers m^6^A methylation at 5’UTR of SLC7A11 mRNA in HCC

**FIGURE 3:**
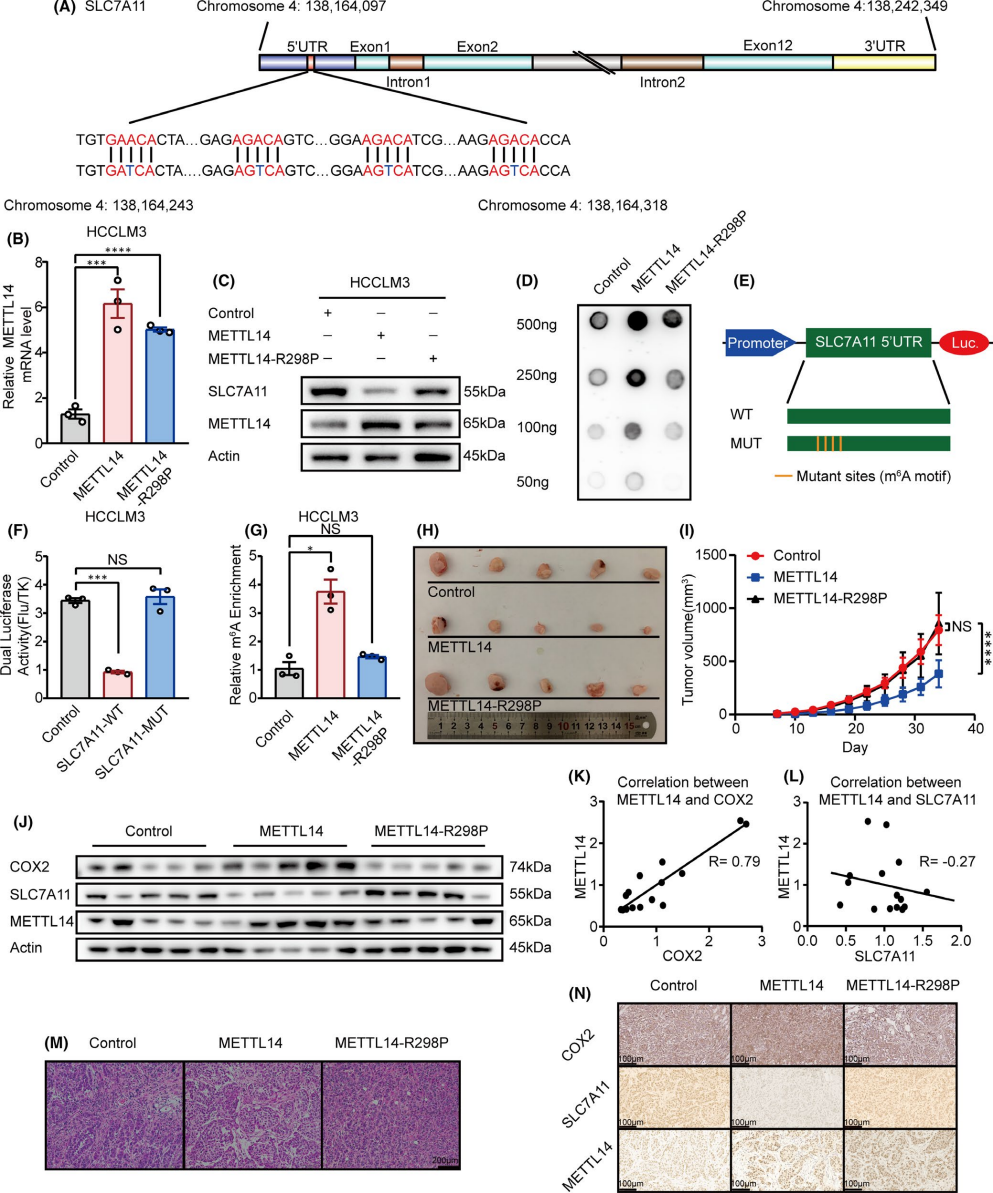
METTL14 triggers m6A methylation of SLC7A11 mRNA at 5’UTR and the anti‐tumour effect of METTL14 is dependent on its methylation activity in HCC. (A) Schematic diagram of SLC7A11 mRNA and the predicted ‘m^6^A’ sites at 5‘UTR are highlighted in red. Base A in the middle of ‘DRACH’ was replaced by T to make the mutant plasmid for luciferase reporter assay. (B&C) The effect of wide‐type METTL14 and METTL14‐R298P mutant on SLC7A11 expression in HCCLM3 cells. The mRNA and protein level of SLC7A11 was detected by qPCR and Western blot, separately. (D) DOT BLOT showed the total m^6^A level HCCLM3 that stably expressed wide‐type METTL14 and METTL14‐R298P mutant. (E) Schematic diagram of the luciferase reporter of SLC7A11. (F) Relative activity of the WT or MUT luciferase reporters based on pGL3‐basic plasmid in METTL14 transfected HCCLM3 cells was determined (normalized to vector control groups). (G) MeRIP analysis followed by RT‐qPCR was applied to assess the m^6^A modification of SLC7A11 in HCCLM3 expressed wide‐type METTL14 or METTL14‐R298P mutant. The enrichment of m^6^A in each group was calculated by m^6^A‐IP/input and IgG‐IP/input. (H) The effect of wide‐type METTL14 and METTL14‐R298P mutant on HCC tumour growth. Nude mice were subcutaneously injected with HCCLM3 cells that stably expressed METTL14, METTL14‐R298P or control vector. Tumour growth was calculated twice every week. (I) Tumour growth curve of stable wide‐type METTL14 or METTL14‐R298P mutant overexpressing HCCLM3 cells (or negative control) in the xenograft model was presented. (J) The expression pattern of COX2, SLC7A11 and METTL14 in the xenograft detected by Western blot. (K) The correlation between METTL14 and COX2 in the xenograft. (L) The correlation between METTL14 and SLC7A11 in the xenograft. M. H&E stained section of three kinds of xenografts. (N) The expression pattern of COX2, SLC7A11 and METTL14 in the xenograft detected by immunohistochemistry. ‘NS’, not significant, *p < 0.05, **p < 0.01, ***p < 0.001, ****p < 0.0001

In order to verify the specific relationship between SLC7A11 and METTL14, we first checked the RNA Base v2.0 (http://www.sysu.edu.cn) and found that there were several potential m^6^A sites within 5'UTR of SLC7A11 mRNA (Figure 3A), which suggested SLC7A11 might be regulated by METTL14 in an m^6^A‐dependent manner.

It has been demonstrated the R298P mutation greatly reduces METTL14 methylation activity.^15^ We therefore established stable METTL14‐R298P expression Huh7 and HCCLM3 cell lines (Figure 3B,C & Figure S1A,S1B).

To identify if the m^6^A modification of SLC7A11 was mediated by METTL14, we first detected the total m^6^A level in negative control group and stable METTL14 overexpression as well as METTL14‐R298P groups through m^6^A dot blot assay. As expected, m^6^A levels were substantially increased with the overexpression of METTL14 but were reduced by R298P mutation in two HCC cell lines (Figure 3D & Figure S1C).

To explore the essence of m^6^A modification on SLC7A11, luciferase reporter assays were conducted with a wild‐type (WT) and a mutant (MUT) plasmid (Figure 3E). For mutant reporter, cytosine bases (C) were designed to replace the adenosine bases (A) in several predicted m^6^A sites to block the effect of m^6^A methylation, while wild‐type reporter contained intact m^6^A sites. As expected, METTL14 overexpression moderately reduced the luciferase activity of wide‐type group, but had little effect on the mutant counterpart (Figure 3F & Figure S1D), indicating that SLC7A11 regulation was under the control of METTL14‐guided m^6^A modification.

Furthermore, the enrichment of m^6^A in SLC7A11 was detected by MeRIP‐qPCR assay. Compared with IgG control group, a significant enrichment of SLC7A11 transcripts was detected in m^6^A‐specific antibody‐treated group. In addition, we observed a dramatic reduced level of SLC7A11 modified by m^6^A following METTL14‐R298P mutant expression (Figure 3G & Figure S1E). Thus, we speculated that METTL14 could affect the overall level of m^6^A, specifically for SLC7A11.

To confirm the role of METTL14 in vivo, tumour xenograft models were constructed by subcutaneously injecting HCC cells (HCCLM3) with either stable overexpression of wide‐type METTL14 or METTL14‐R298P mutant into nude mice. We found that wide‐type METTL14 overexpression repressed tumorigenesis with prominently lower tumour volumes compared with control group. Meanwhile, forced expression of METTL14‐R298P mutant lost the tumour‐repressive role in xenograft mice (Figure 3H,3I). Consistently, compared to control and METTL14‐R298P mutant group, METTL14‐overexpresssion group exhibited lower level of SLC7A11 and higher level of COX2, which is the golden index of ferroptosis^48^, ^49^ in the xenografts (Figure 3J). The negative correlation between METTL14 and SLC7A11, and the positive correlation between METTL14 and COX2 in the xenografts were also shown (Figure 3K,L). Additionally, the H&E staining of tumour sections was indicated as tumour structures (Figure 3M). The expression of METTL14, SLC7A11 and COX2 in xenografts tumour sections was further investigated by immunohistochemistry. METTL14 overexpression group exhibited lower expression of SLC7A11 and higher expression of COX2 compared to the other two groups (Figure 3N).

In summary, these data suggested that METTL14 performed a tumour‐suppressive function via targeting SLC7A11 in an m^6^A‐dependent manner in HCC.

### 3.4 METTL14‐induced SLC7A11 mRNA decay is m^6^A‐YTHDF2‐dependent

**FIGURE 4:**
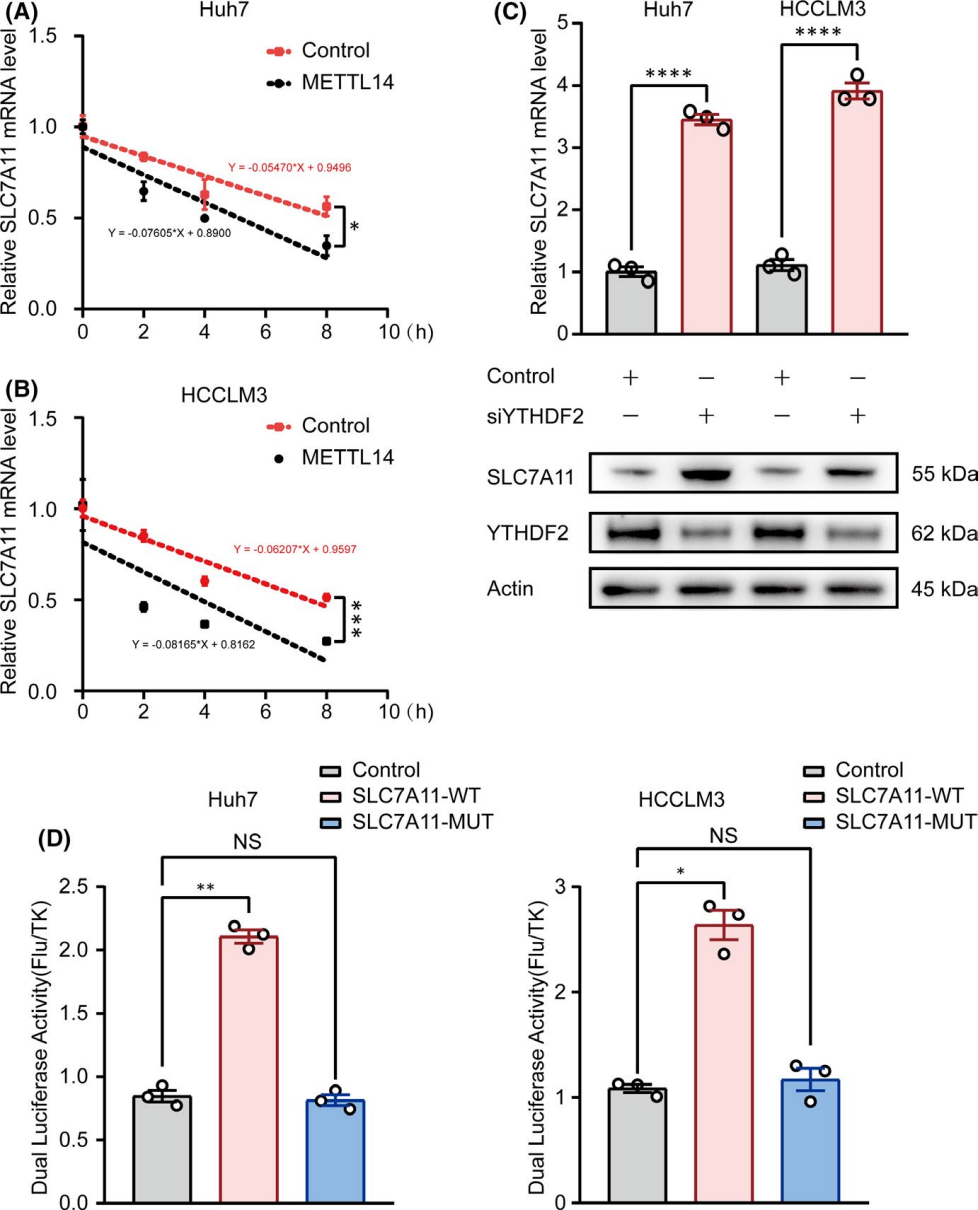
m6A methylated 5’UTR regulates mRNA degradation of SLC7A11. (A&B) The mRNA decay rate was determined in Huh7 and HCCLM3 cells after treatment with Actinomycin D (normalized to 0h). (C) RT‐qPCR and Western blot showed the impact of silencing YTHDF2 on SLC7A11 mRNA and protein levels in Huh7 and HCCLM3. (D) Relative activity of the WT or MUT luciferase reporters in Huh7 and HCCLM3 cells transfected with siYTHDF2 was determined (normalized to vector control groups). ‘NS’, not significant, *p < 0.05, **p < 0.01, ***p < 0.001, ****p < 0.0001

It is essential to figure out the readers of SLC7A11, since m^6^A‐modified mRNA transcripts depend on the reader proteins to functionally participate in biological processes. Previously, we demonstrated METTL14 overexpression remarkably downregulated SLC7A11 mRNA in both Huh7 and HCCLM3 cell lines (Figure 2J). Next, we tested if m^6^A modification affects the mRNA stability of SLC7A11. qPCR showed that overexpression of METTL14 significantly enhanced SLC7A11 mRNA degradation in the presence of Actinomycin D (Figure 4A,4B).

YTHDF2 (YTH domain family 2), a recognized m^6^A reader protein, has been demonstrated to regulate the mRNA stability. Strikingly, we found knockdown of YTHDF2 substantially increased SLC7A11 expression at both mRNA and protein levels (Figure 4C), which indicated the potential role of YTHDF2 in SLC7A11 modulation. Furthermore, luciferase reporter assays were performed with plasmid containing the wild‐type (WT) or mutant (MUT) SLC7A11 as described previously. As expected, knockdown of YTHDF2 dramatically increased the luciferase activity of wide‐type group, but had little effect on the mutant group (Figure 4D). Taken together, these data indicated that m^6^A ‐YTHDF2 conducts METTL14‐induced SLC7A11 mRNA degradation.

### 3.5 Knockdown of SLC7A11 stimulates ferroptosis and exhibits an anti‐tumour effect in HCC

**FIGURE 5:**
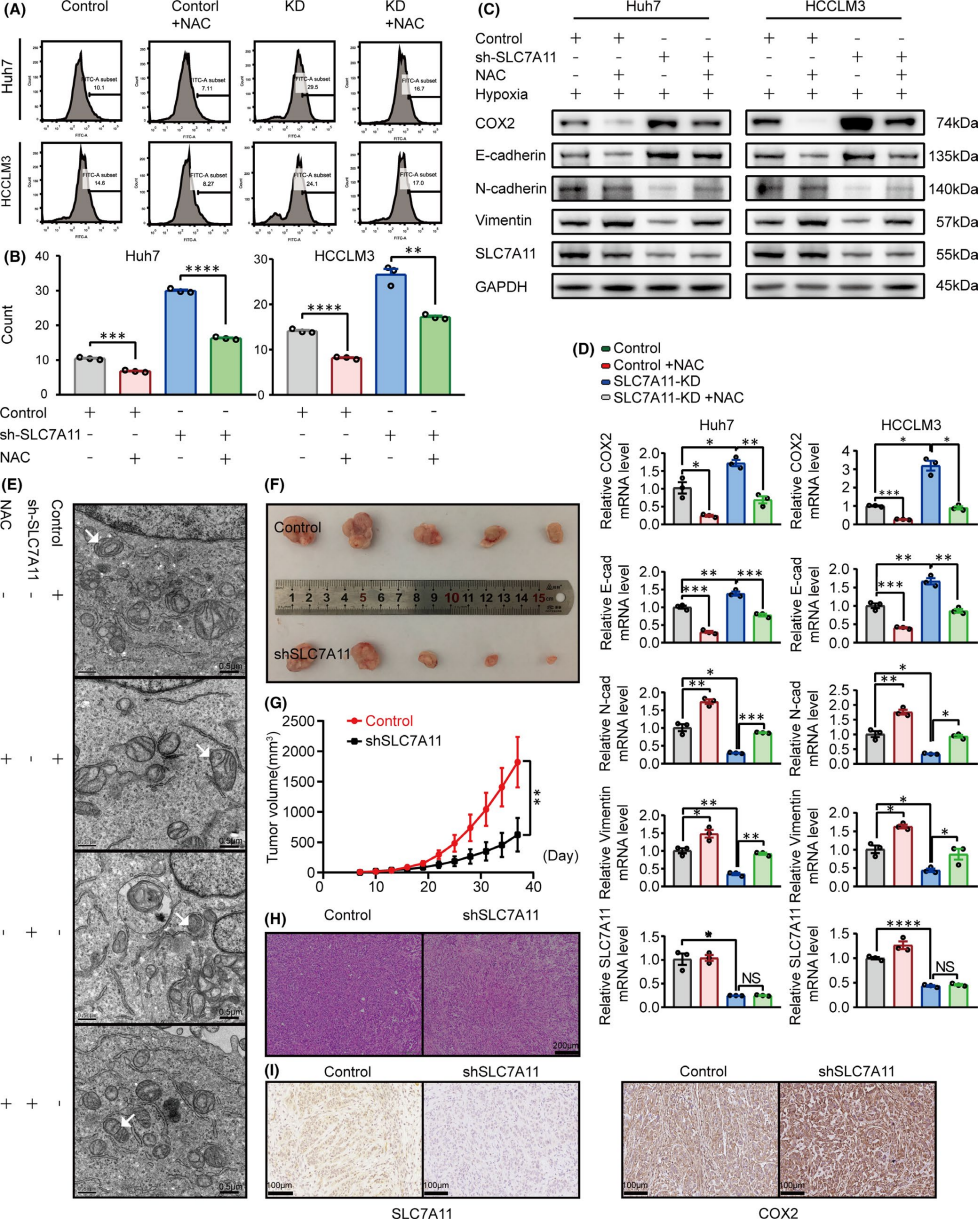
Knockdown of SLC7A11 stimulates ferroptosis and exhibits an anti‐tumour effect in HCC. A&B. ROS detection was performed by flow cytometry according to the manufacturers’ instruction (A) and quantified (B). The Huh7 and HCCLM3 cells were treated with or without 10mM concentrations of NAC for 36 h. (C&D) The protein and mRNA levels of EMT related E‐cadherin, N‐cadherin and Vimentin were detected by Western blot and qPCR in Huh7 and HCCLM3 cells, respectively. Cells were treated with or without 10 mM concentrations of NAC for 36 h. (E) The morphological alterations of mitochondria treated with/without NAC or shSLC7A11 in HCCLM3 cells were detected by electronic microscopy. The white arrows referred to typical mitochondria. (F) The effect of SLC7A11 knockdown on the tumour growth. G. Tumour volume was monitored during the time course of 5 weeks. (H) H&E stained section of three kinds of xenografts described above. (I) The expression pattern of SLC7A11 and COX2 in the xenograft detected by immunohistochemistry. ‘NS’, not significant, *p < 0.05, **p < 0.01, ***p < 0.001, ****p < 0.0001

To address if SLC7A11 inhibition can mimic the tumour‐suppressive function of METTL14, we first detected the effect of SLC7A11 knockdown on ferroptosis induction. As Figure 5A,B shown, shSLC7A11 strongly stimulated ROS production, while the ROS scavenger N‐acetyl‐L‐cysteine (NAC) dramatically blocked the shSLC7A11‐induced ROS accumulation as well as EMT reversion (Figure 5C,D) in both Huh7 and HCCLM3 cells. Moreover, NAC treatment potently abrogated the shSLC7A11 induced mitochondria shrinking detected by electronic microscopy. Compared to the shSLC7A11 group, the mitochondria in shSLC7A11 plus NAC group exhibited relatively more intact membrane and larger size (Figure 5E). In addition, the same tendency was observed in vivo by nude mice. Compared to the control group, downregulation of SLC7A11 significantly inhibited tumour growth (Figure 5F,G). The H&E staining of tumour sections was indicated as tumour structures (Figure 5H). Furthermore, immunohistochemistry analysis showed that the shSLC7A11 group had a higher expression of COX2 (Figure 5I). These data verified the anti‐tumour effect by inhibiting of SLC7A11 in HCC cells.

### 3.6 Ectopic expression of SLC7A11 abrogates METTL14‐induced tumour‐suppressive effect under hypoxia in HCC

**FIGURE 6:**
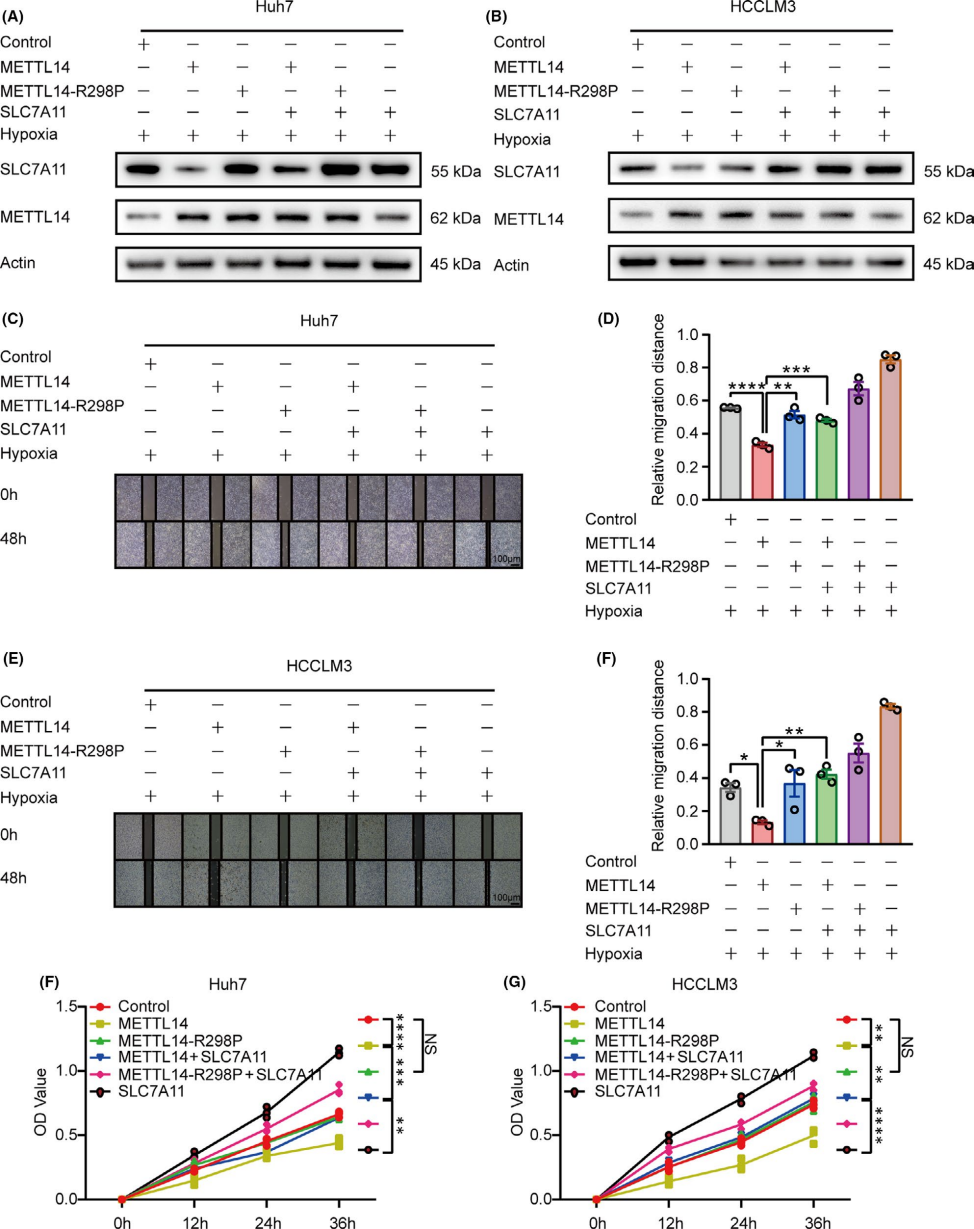
Ectopic expression of SLC7A11 abrogates METTL14‐induced tumour‐suppressive effect under hypoxia in HCC. A&B. Western blot showed the expression pattern of METTL14 and SLC7A11 in Huh7 and HCCLM3 cells. Cells stably expressed wide‐type METTL14 or R298P mutant were overexpressed (transfected) with SLC7A11. C‐F. Wound healing showed the migration rate of Huh7 and HCCLM3 cells transfected with or without SLC7A11 plus METTL14‐control, METTL14 overexpression or METTL14‐R298P under hypoxic condition, respectively. G&H. CCK‐8 assay illustrated the cell viability of Huh7 and HCCLM3 cells transfected with or without SLC7A11 plus METTL14‐control, METTL14 overexpression or METTL14‐R298P under hypoxia, respectively. ‘NS’, not significant, *p < 0.05, **p < 0.01, ***p < 0.001, ****p < 0.0001

We further examined whether inhibition of SLC7A11 contributes to the anti‐tumour effects of METTL14. As Figure 6A,B shown, under the hypoxic environment, wide‐type METTL14 but not the METTL14‐R298P mutant strongly inhibited SLC7A11 expression, while overexpression of SLC7A11 effectively abolished the downregulation of SLC7A11 induced by METTL14 in both HCC cell lines. As expected, the resulting SLC7A11 overexpression obviously abrogated the METTL14‐induced cell migration inhibition (Figure 6B–F). Furthermore, SLC7A11 overexpression also significantly blocked the cell viability induced by METTL14 in both Huh7 and HCCLM3 cells (Figure 6G,H). Taken together, all these data suggest that SLC7A11 is potently involved in METTL14‐regulated growth and migration.

## 4 DISCUSSION

**FIGURE 7:**
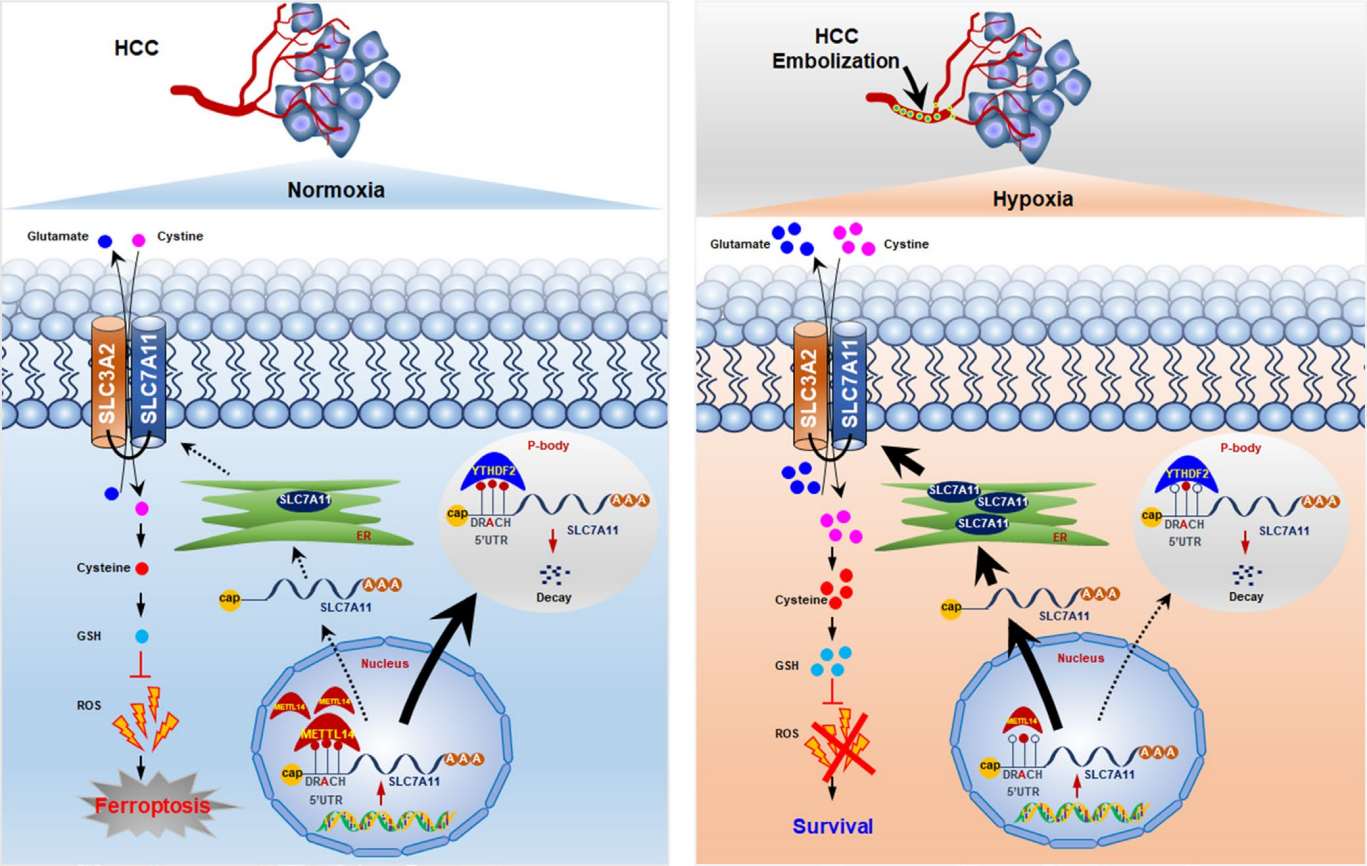
Model of METTL14‐induced YTHDF2/SLC7A11/ROS axis mediated ferroptosis under hypoxia triggered by embolization in HCC cells

Herein, we reported that hypoxia induced suppression of METTL14 which played a tumour‐suppressive role in HCC via induction of ferroptosis. METTL14 targeted m^6^A methylation at 5' UTR of SLC7A11 mRNA and enhanced SLC7A11 mRNA degradation in an m^6^A‐YTHDF2‐dependent manner (Figure 7). Specifically, under normoxia condition, METTL14 targeted m^6^A methylation at 5' UTR of SLC7A11 mRNA, and the m^6^A‐modified SLC7A11 mRNA was recognized by the ‘reader’ YTHDF2 followed by sending to P‐body for degradation. The SLC7A11 depletion would result in decreased import of cystine, cysteine and GSH accumulation, which eventually stimulated ROS production and induced ferroptosis. Conversely, hypoxia triggered by interventional embolization would inhibit METTL14 in a HIF‐1α–dependent manner, which subsequently blocked the METTL14/YTHDF2/SLC7A11/ROS axis mediated ferroptosis and promoted HCC progression.

Ferroptosis which is a newly discovered way of programmed cell death has been demonstrated to be related to iron metabolism and reactive oxygen species (ROS).^46^, ^50^ ROS are broadly defined as chemically reactive molecules, which mainly include superoxide anion (O_2_^−^), singlet oxygen (^1^O_2_), hydroxyl radical (OH) and hydrogen peroxide (H_2_O_2_).^51^ Either normal cells or cancer cells have their own means to keep the ROS balance.^52^ However, if the balance is broken, cells are turning to another totally different status. ROS can cause DNA damage and induces the DNA mutagenesis.^53^ Currently, the molecular mechanisms by which ROS affects tumour pathogenesis are still poorly understood.

It is well‐known that migration is usually driven by EMT.^54^ In this study, we did observe that silence of SCL7A11, a key member of ferroptosis signalling pathway, strongly upregulated E‐cadherin and downregulates N‐cadherin and Vimentin at both mRNA and protein levels, which provided a reasonable evidence that silence of SLC7A11 might inhibit migration of HCC cells. Under hypoxia, we used N‐acetyl‐L‐cysteine (NAC), an ROS inhibitor,^55^ to explore whether the change of EMT by silence of SLC7A11 would be rescued. Although ROS increased with the silence of SLC7A11, ROS can be eliminated to some extent in both groups. On the contrary, E‐cadherin decreased, N‐cadherin and Vimentin increased under the treatment of NAC at both mRNA and protein levels. Collectively, all the results together with the reports that ferroptosis is closely correlated to drug resistance and synthetic lethality in multiple tumours^30^, ^56^ strongly suggest that ferroptosis plays a key role in HCC tumorgenicity. However, few findings of epigenetic regulation of ferroptosis have been obtained.

m^6^A methylation, which is the most common modification of RNA methylation, has been implicated in regulation of many biological processes, including tumour initiation and progression.^57^ However, little is known about the crosstalk between ferroptosis and RNA methylation. Surprisingly, we found several potential modification sites of m^6^A methylation within the 5’UTR of SLC7A11 mRNA at the RNA Base v2.0 (http://www.sysu.edu.cn), which provided the possibility that SLC7A11 might be modified by m^6^A‐related enzymes, ‘Writer’, ‘Eraser’ and ‘Reader’.

METTL14 is one of the ‘Writer’ members of m^6^A modification. Interestingly, both bioinformatic analysis and Western blot showed that METTL14 was downregulated in HCC and was inversely related to SLC7A11 expression, which gave us a clue to hypothesize that METTL14 might negatively regulate SLC7A11. Therefore, we chose two cell lines (Huh7 and HCCLM3) to establish the METTL14‐overexpressed stable cell lines. As expected, METTL14 not only downregulated SLC7A11, but also inhibited the migration and proliferation of HCC cells. Our results indicate that the anti‐tumour effect induced by METTL14 might be intervened by SLC7A11. We aimed at finding the concrete mechanism that how did METTL14 downregulate SLC7A11. As described above, if the residue of Arginine 298 is replaced by Proline, the function of RNA methyltransferase of METTL14 would be abolished. We established METTL14‐control, METTL14‐overexpression and the METTL14‐R298P stable cell lines. As expected, wide‐type METTL14 can dramatically inhibit SLC7A11 expression at both mRNA and protein levels. However, R298P mutant failed to affect the SLC7A11 expression. On the contrary, SLC7A11 had no effect on METTL14 expression. Under these circumstances, wound healing assays showed that METTL14 inhibited migration via downregulation of SLC7A11, and overexpression of SLC7A11 prevented the METTL14‐ induced migration inhibition. Dual‐luciferase reporter assay further illustrated METTL14 could directly regulate SLC7A11. All the data indicate that METTL14 targets SLC7A11 in an m^6^A‐dependent manner.

The function of YTHDF2 has been confirmed to drive the mRNA degradation.^58^ Since METTL14 negatively regulates SLC7A11, we hypothesized that YTHDF2 was the key ‘reader’ that induced SLC7A11 mRNA decay. The group of METTL14 overexpression showed a decreased mRNA half‐life than that of METTL14‐control, which implied that YTHDF2 might be the major ‘Reader’ of SLC7A11. As expected, YTHDF2 reduction can remarkably upregulate SLC7A11 at both mRNA and protein levels, which was further confirmed by dual‐luciferase reporter assay. Our data clearly support that YTHDF2 contributes to the decay of m^6^A‐modified SLC7A11 mRNA. Interestingly, a recent report demonstrated that YTHDC2 interacted with m^6^A methylated SLC7A11 mRNA at 3’UTR.^59^ However, our study revealed METTL14 could target the 5’UTR of SLC7A11 mRNA to induce its degradation via YTHDF2‐dependent pathway. Our findings together with others strongly suggest that ferroptosis mediated by SLC7A11, the key component of system Xc^−^ complex, is accurately regulated by m^6^A modification.

The function of hypoxia‐induced HIF‐1α has been widely investigated in HCC progression.^60^ Zhang et. al. has reported that hypoxic stress in the HCC cells promoted YAP binding to HIF‐1α in the nucleus and sustained HIF‐1α protein stability to bind to PKM2 gene and directly activated PKM2 transcription to accelerate glycolysis^61^; Zhou et. al. showed that HIF‐1α activated the transcription of lncRNA RAET1K to modulate hypoxia‐induced glycolysis in hepatocellular carcinoma cells via miR‐100‐5p.^62^ Furthermore, the effect of hypoxia on ferroptosis has also been studied. Li et. al. demonstrated that carbonic anhydrase 9 (CA9) conferred resistance to ferroptosis in malignant mesothelioma under hypoxia.^63^ In their study, they provided the evidence that CA9 played a key role in equilibrating among hypoxia, iron metabolism and redox regulation in MM cells, and subsequently promoted cell survival. However, the exact mechanisms underlying HIF‐1α‐regulated ferroptosis are still unclear. In our study, we revealed that hypoxia‐induced HIF‐1α could block ferroptosis and promote HCC survival via METTL14/YTHDF2 pathway, suggesting that targeting HIF‐1α/METTL14/YTHDF2 signal axis might have a synergistic effect to HCC interventional treatment.

Overall, our data clearly suggested that the METTL14‐induced ferroptosis could be dramatically abolished in hypoxic environment, which contributed to HCC progression. These findings identified the HIF‐1α/METTL14/YTHDF2/SLC7A11 axis as a promising therapeutic target for the HCC treatment. Further studies are still needed to fully investigate the efficacy of interfering HIF‐1α/METTL14/YTHDF2/SLC7A11 axis with small molecule or gene therapy in clinic to better advance the HCC interventional embolization treatment.

## CONFLICT OF INTEREST

The authors declare no conflict of interest.

## AUTHOR CONTRIBUTIONS

**Zhuoyang Fan:** Data curation (lead); Validation (lead); Writing‐original draft (lead); Writing‐review & editing (lead). **Guowei Yang:** Data curation (equal); Investigation (equal); Resources (equal); Visualization (equal). **Wei Zhang:** Data curation (equal); Investigation (equal); Resources (equal); Visualization (equal). **Qian Liu:** Data curation (equal); Investigation (equal); Resources (equal); Visualization (equal). **Guangqin Liu:** Formal analysis (supporting); Software (supporting). **Ligang Xu:** Methodology (supporting); Software (supporting). **Pingping Liu:** Data curation (supporting). **Jianhua Wang:** Conceptualization (supporting); Supervision (supporting). **Zhiping Yan:** Conceptualization (supporting); Validation (supporting). **Hong Han:** Conceptualization (equal); Resources (equal); Supervision (equal); Writing‐original draft (equal). **Rong Liu:** Conceptualization (equal); Resources (equal); Supervision (equal); Writing‐original draft (equal); Writing‐review & editing (equal). **Minfeng Shu:** Conceptualization (lead); Supervision (lead); Writing‐review & editing (lead).

## Supporting information

Fig S1

Supplementary Material

## Data Availability

The data that support the findings of this study are available on request from the corresponding author. The data are not publicly available due to privacy or ethical restrictions.
