# Supplementary figures and images for: Hypoxia blocks ferroptosis of hepatocellular carcinoma via suppression of METTL14 triggered YTHDF2‐dependent silencing of SLC7A11
Source: J Cell Mol Med. 2021 Oct 5;25(21):10197–212. doi: 10.1111/jcmm.16957 (PMC8572766; doi:10.1111/jcmm.16957)

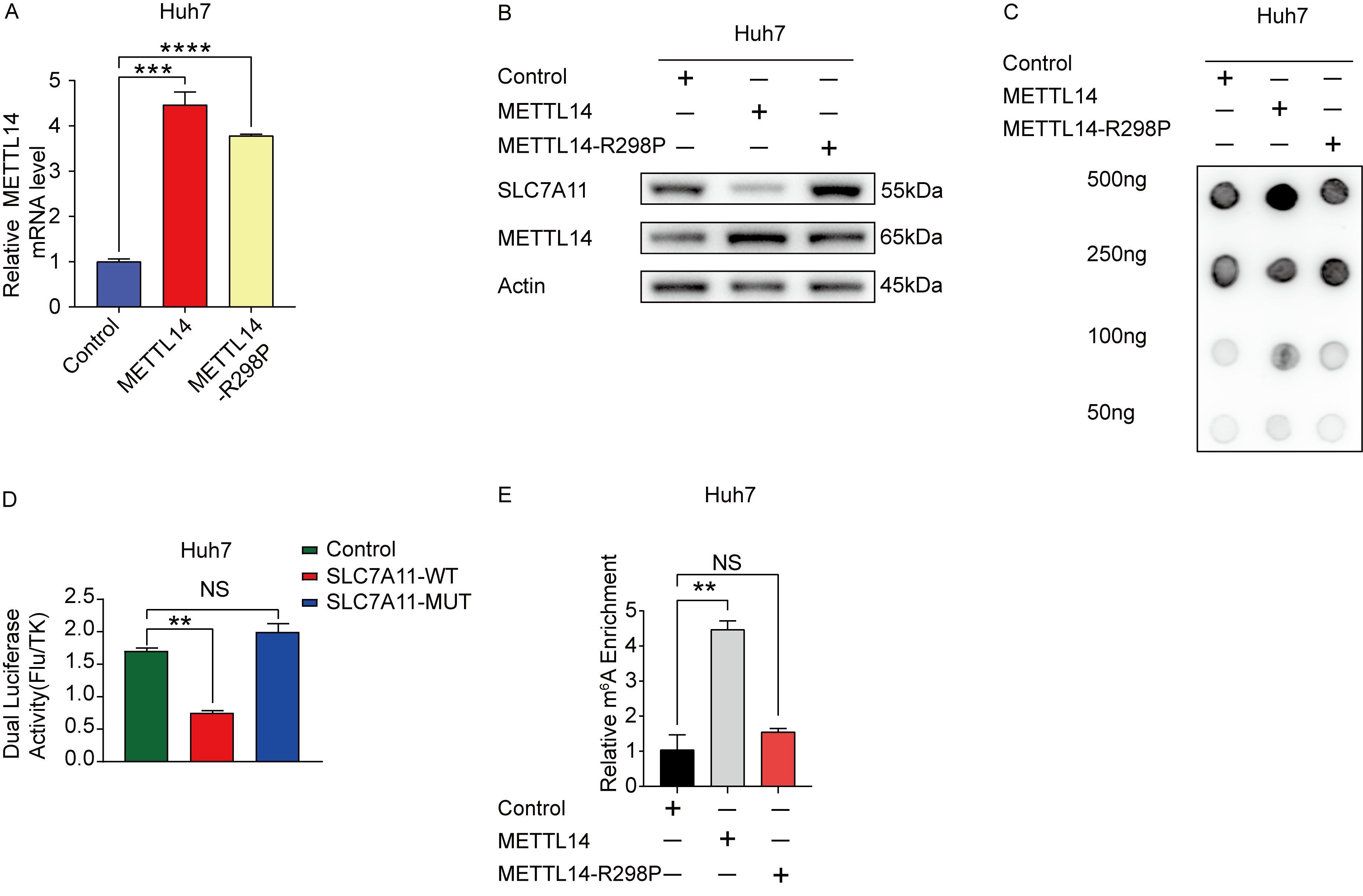

Supplement: Supplementary file 1 — Fig S1 [file JCMM-25-10197-s002.png]
