# Supplementary material for: Hypoxia blocks ferroptosis of hepatocellular carcinoma via suppression of METTL14 triggered YTHDF2‐dependent silencing of SLC7A11
Source: J Cell Mol Med. 2021 Oct 5;25(21):10197–212. doi: 10.1111/jcmm.16957 (PMC8572766; doi:10.1111/jcmm.16957)
Supplement: Supplementary file 2 — Supplementary Material [file JCMM-25-10197-s001.docx]

**Supplementary Figure 1.**

**Figure S1. METTL14 triggers m^6^A methylation of SLC7A11 mRNA in Huh7 cells.**

A&B. The effect of wide type METTL14 and METTL14-R298P mutant on SLC7A11 expression in Huh7 cells. The mRNA and protein level of SLC7A11 was detected by qPCR and Western blot, respectively.

C. m^6^A DOT BLOT showed the total m^6^A level in Huh7 cells that stably expressed wide type METTL14 and METTL14-R298P mutant.

D. Relative activity of the WT or MUT luciferase reporters based on pGL3-basic plasmid in METTL14 transfected Huh7 cells was determined (normalized to vector control groups).

E. MeRIP analysis followed by RT-qPCR was applied to assess the m^6^A modification of SLC7A11 in Huh7 cells expressed wide type METTL14 or METTL14-R298P mutant. The enrichment of m^6^A in each group was calculated by m^6^A-IP/input and IgG-IP/input.
